# AK7-deficiency reversal inhibits ccRCC progression and boosts anti-PD1 immunotherapy sensitivity

**DOI:** 10.18632/aging.206006

**Published:** 2024-07-05

**Authors:** Yigang Jin, Minjie Chen, Fei Chen, Zhaofeng Gao, Xiaoping Li, Lingyu Hu, Dandan Cai, Siqi Zhao, Zhengwei Song

**Affiliations:** 1 Department of Urology, The Second Affiliated Hospital of Jiaxing University, Jiaxing, Zhejiang, China; 2 Department of Surgery, The Second Affiliated Hospital of Jiaxing University, Jiaxing, Zhejiang, China

**Keywords:** adenylate kinase 7, ccRCC, immunotherapy, anti-PD1

## Abstract

Clear cell renal cell carcinoma (ccRCC) is a common kidney cancer with subtle early symptoms, high recurrence rates, and low sensitivity to traditional treatments like radiotherapy and chemotherapy. Identifying novel therapeutic targets is critical. The expression level of adenylate kinases 7 (AK7) in ccRCC was examined by the TCGAportal and UALCAN databases. The effect of AK7 on proliferation, invasion and migration of ccRCC cell lines was evaluated by cell assay. The correlation between AK7 expression and prognosis, as well as its direct relationship with immunotherapy efficacy, was analyzed using CANCERTOOL and Kaplan-Meier plotter data. Moreover, the TISIDB database was used to study the relationship between AK7 and immune markers. The effect of overexpressed AK7 combined with PD1 monoclonal antibody on ccRCC was evaluated in animal experiments. The results showed that low level of AK7 expression was observed in ccRCC tissues. The expression of AK7 can regulate the proliferation, invasion, and migration of human ccRCC cell lines. The level of AK7 expression was associated with OS of ccRCC patients. This was potentially due to the negative connection between AK7 expression and CD8+ T cell depletion, indicating that immunotherapy might be less effective in individuals with low AK7 expression. Conversely, augmenting AK7 demonstrated an enhanced effectiveness of anti-PD1 therapy. The findings of our research strongly indicated that AK7 could serve as both a prognostic indicator and therapeutic target for patients with ccRCC. Moreover, the overexpression of AK7 combined with anti-PD1 held promising potential as a therapeutic approach for treating ccRCC.

## INTRODUCTION

Kidney carcinoma is a prevalent and significant kidney disorder, with its incidence steadily rising in recent years, now representing approximately 2.2% of all cancer cases [1]. RCC is the predominant form of renal carcinoma, comprising over 90% of all renal cancer types. This type of cancer is categorized into three different subtypes. The most prevalent one is ccRCC, constituting 80–90% of RCC cases. The other subtypes are papillary cell carcinoma (15%) and chromophobe cell carcinoma (5%) [2]. Despite surgery being the primary treatment for RCC, a notable proportion of individuals (20–40%) may still encounter the recurrence and spread following surgery [3]. Currently, the effectiveness of traditional clinical treatments such as radiotherapy or cytotoxic chemotherapy drugs for treating RCC is quite limited, with only a subset of patients deriving benefits from it. Currently, the advancement and evolution of targeted drugs aimed at mammalian target of rapamycin (mTOR) and receptor tyrosine kinase (RTK) signaling have led to therapeutic progress, but the emergence of medication tolerance is a major dilemma [3–8].

In the realm of cancer treatment, immunotherapy has recently come to the forefront as a promising area, with Immune Checkpoint Inhibitors (ICIs) such as anti-PD1, anti-programmed death ligand-1 (PD-L1), and anti-cytotoxic T-lymphocyte antigen-4 (CTLA-4) in treating ccRCC [9–11]. These inhibitors have shown effectiveness either as standalone therapies or in combination with other treatment options [12, 13]. However, similar to other tumor patients, a considerable number of ccRCC patients experience primary or secondary drug resistance during treatment [14]. These therapeutic limitations have stimulated additional investigations exploring different combinations and innovative approaches to enhance the effectiveness of immunotherapy [15–17]. Adenylate kinases, small and frequently monomeric enzymes, are ubiquitous in all living organisms and serve a vital function in energy metabolism [18, 19]. The human adenylate kinase genes are situated in varied chromosomes, and their distribution within organs and intracellular localization elucidate their dysregulation in several disorders. Previous studies have reported that the participation of AKs in adenosine triphosphate (ATP) control is associated with additional intracellular mechanisms, including stress response, circadian rhythms, and the pathological transformation of cancer cells [20–22]. The primary adenylate kinases in this network of inflammation regulation are cytoplasmic isozyme 1 and mitochondrial isozyme 2 [23–27]. Cytoplasmic isozyme 5 is associated with marginal encephalitis [28, 29]. Research has revealed that neuroblastoma or glioma may cause impairment in mitochondrial isoenzymes 2 and 4 [18, 30]. The AK7 gene is the only cytoplasmic AK gene in the AK family and located at 14q32.2 on chromosome 14 (NCBI Gene ID: 122481) [31, 32]. Previous studies have indicated that mutations in the AK7 gene could be linked to primary ciliary dyskinesia (PCD) and primary male infertility [31–34]. Furthermore, research in the field of cancer has reported that it may serve as a potential prognostic indicator of overall cancer survival [35]. The study observed a decrease in AK7 expression in ccRCC and revealed a correlation between AK7 expression, patient prognosis and immunotherapy effect. These findings suggested that AK7 could function as a predictive indicator of prognosis for ccRCC and may be a target for enhancing the sensitivity of anti-PD1 therapy.

## MATERIALS AND METHODS

### Analysis of AK7 expression

TCGAportal (http://www.tcgaportal.org), UALCAN (http://ualcan.path.uab.edu/), and HPA (https://www.proteinatlas.org/) databases were employed to examine the levels of AK7 in different tissue adjacent to tumor and cancer. The databases were further used to make a comparison of the expression of AK7 in ccRCC individuals with various stages, grades, subtypes, lymph node metastasis and race.

### Cell culture and transfection

Cells were cultured in a 5% CO_2_ chamber at 37°C with RFMI-1640 (Gibco, USA) containing 10% FBS (NEWZERUM, Newzerum, Christchurch, New Zealand) and 1% P-S. si-AK7 and si-NC were produced by KeyGEN BioTECH (Jiangsu, China). We utilized the Lipofectamine 2000 (Invitrogen, USA) and Opti-MEM (Gibco, USA) for transfection process. The cells were collected 2 d after transfection for subsequent analysis. The specific target sequence of si-AK7 is: hAK7 si-1 sense: CCAAGGACUUAACGCAAGAUUTT, hAK7 si-1 antisense: AAUCUUGCGUUAAGUCCUUGGTT; hAK7 si-2 sense: GCAGAAUGUUUCCCUUUGAUATT, hAK7 si-2 antisense: UAUCAAAGGGAAACAUUCUGCTT; hAK7 si-3 sense: AGGAGGCAUGUUACACACAUUTT, hAK7 si-3 antisense: AAUGUGUGUAACAUGCCUCCUTT. For overexpression studies, we inserted the cDNA of AK7 into pcDNA3.1. We transfected the cells, with the control transfection agent being the plasmid vector pcDNA3.1. For overexpression experiments in mice, we enlisted KeyGEN BioTECH (Jiangsu, China) to build a lentivirus capable of inducing AK7 overexpression. We seeded 1 × 10^5^ cells/well and incubated them in 2 mL of medium for a whole day. Subsequently, the medium was exchanged for 1 mL, to which the correct quantity of virus and 40 μL of polyburene (Sigma-Aldrich, USA) were introduced. Following a 12–16 hours incubation period, the cells were transferred to standard growth medium. To assess the transfection efficiency, a qRT-PCR assay was conducted.

### Constructing and transfecting process of plasmids

The control plasmid pcDNA3 and the pcDNA3-AK7 plasmid for AK7 overexpression were supplied by KeyGEN BioTECH (Jiangsu, China). The overexpression of the pcDNA3-AK7 plasmid involved transfection with pcDNA3.1 (+), encompassing the full-length cDNA gene of human AK7, and subsequent Hind III and EcoR I recognition processes. The cells were seeded in plates and incubated for a whole day. The cells were transfected by 2 μg plasmid in SFM using DNA FectinTMPlus (Abcam, Canada). Following 12–16 h, the SFM was replaced by CM. The cells were then ready for the subsequent experimental protocols.

### RNA extraction and qRT-PCR

The total RNA was isolated using the RNA extraction kit (Tiangen, China). The qRT-PCR analysis was performed using the SYBR Green Master Mix kit (YEASHEN, China). Glyceraldehyde-3-phosphate dehydrogenase (GAPDH) was employed as the reference gene. The data were examined by 2^−ΔΔCt^. The primer sequences for AK7:5′-CCTACAGCAGCGGAAACATC-3′ (Forward) and 5′-GCCCACAATCTGGAATGTGC-3′ (Reverse).

### Cell proliferation assay

786-O cells and AKI-1 cells were inoculated into separate 96-well plates. In each well, 1000 cells were seeded in medium and CCK-8 solution (RiboBio, Guangzhou, China). The absorbance of the cells at 450 nm was assessed by a microplate reader at 0, 1, 2, and 3 d following the initiation of the culture (Synergy, USA).

### Transwell migration and invasion assays

To the upper chamber, 200 μL of SF-RPMI 1640 medium was added, while the lower chamber received 700 μL of RPMI 1640 medium contained 10% fetal bovine serum (FBS). Each well was inoculated with 20,000 cells. To evaluate the cell invasion ability, the Transwell cavity (Corning, USA) was coated with a Matrigel mixture (BD Biosciences, USA). After incubating for 1 d in a standard incubator, the upper cavity was extracted, and the SFM was pumped. The cells were then fixed with 4% PFA, followed by staining with crystal violet (China Kagan). The cells were subsequently rinsed with PBS. Finally, the cell lines were observed and counted under a microscope.

### Clone forming assays

Normal and transfected cells were transfected in plate at 1000 cells/well, and subsequently incubated in RPMI 1640 medium containing 10% FBS. After 10 days, the cells were treated with methanol for fixation and then subjected to GIMSA staining. The colonies were visualized and counted.

### Survival analysis

We utilized the Kaplan-Meier plotter (http://kmplot.com/analysis/index.php?p=background) database to assess the connection between the various genes (mRNA, miRNA, protein and DNA) expression and survival outcomes across 35000 samples spanning 21 tumor types. The aforementioned database was employed to investigate the link between AK7 expression levels and prognosis among individuals affected by ccRCC. We leveraged the Kaplan-Meier survival plots to examine and contrast diverse patient cohorts, and subsequently computed hazard ratios and log-rank *P*-values with 95% CI.

### Immune-related analysis

TISIDB (http://cis.hku.hk/TISIDB/index.php), a platform that integrates diverse types of heterogeneous data, is applied for studying the interplay between carcinoma and the immunological system [36]. Here, we used it to examine the Spearman correlation between AK7 expression and some immunomodulators.

### Animal models

Animal tests have been evaluated and endorsed by the Ethics Committee of Jiaxing University, and adhere to pertinent regulations concerning animal ethics and welfare.

Twenty male C57BL/6 mice were separated into four sets at random: OE-NC set, OE-AK7 set, OE-NC+anti-PD1 set and OE-AK7+anti-PD1 set. We used 2 × 10^6^ Renca cells (OE-NC and OE-AK7) to construct a tumor-bearing model of corresponding C57BL/6 mice by subcutaneous injection. On day 8, mice (OE-NC+anti-PD1 set and OE-AK7+anti-PD1 set) were injected intraperitoneally with 6.6 mg/kg of anti-PD1, or the same amount of PBS (OE-NC group and OE-AK7 group), and then injected every 4 days. The mice's general conditions were monitored daily both before and after the study. The vernier calipers were employed to quantify the longitudinal diameter a (mm) and transverse diameter b (mm) of tumor on the body's surface, and then to determine the volume of the tumor using the formula V = ab^2^/2. By measuring the volume of the tumor every 4 days, the final tumor growth curve is generated. On the 20^th^ day, the mice were euthanized, and data on the quality of the tumor were gathered for subsequent analysis.

### Statistical analysis

The mathematical analysis was conducted using GraphPad Prism 9.0. Data were presented as mean ± SD. The two-tailed *t*-test was utilized to examine two independent samples. ANOVA was employed to assess diversity among groups. Prognostic analysis was conducted using the Kaplan-Meier plotter. Statistical significance was *P* < 0.05.

## RESULTS

### The expression of AK7 was decreased in ccRCC

**Figure 1:**
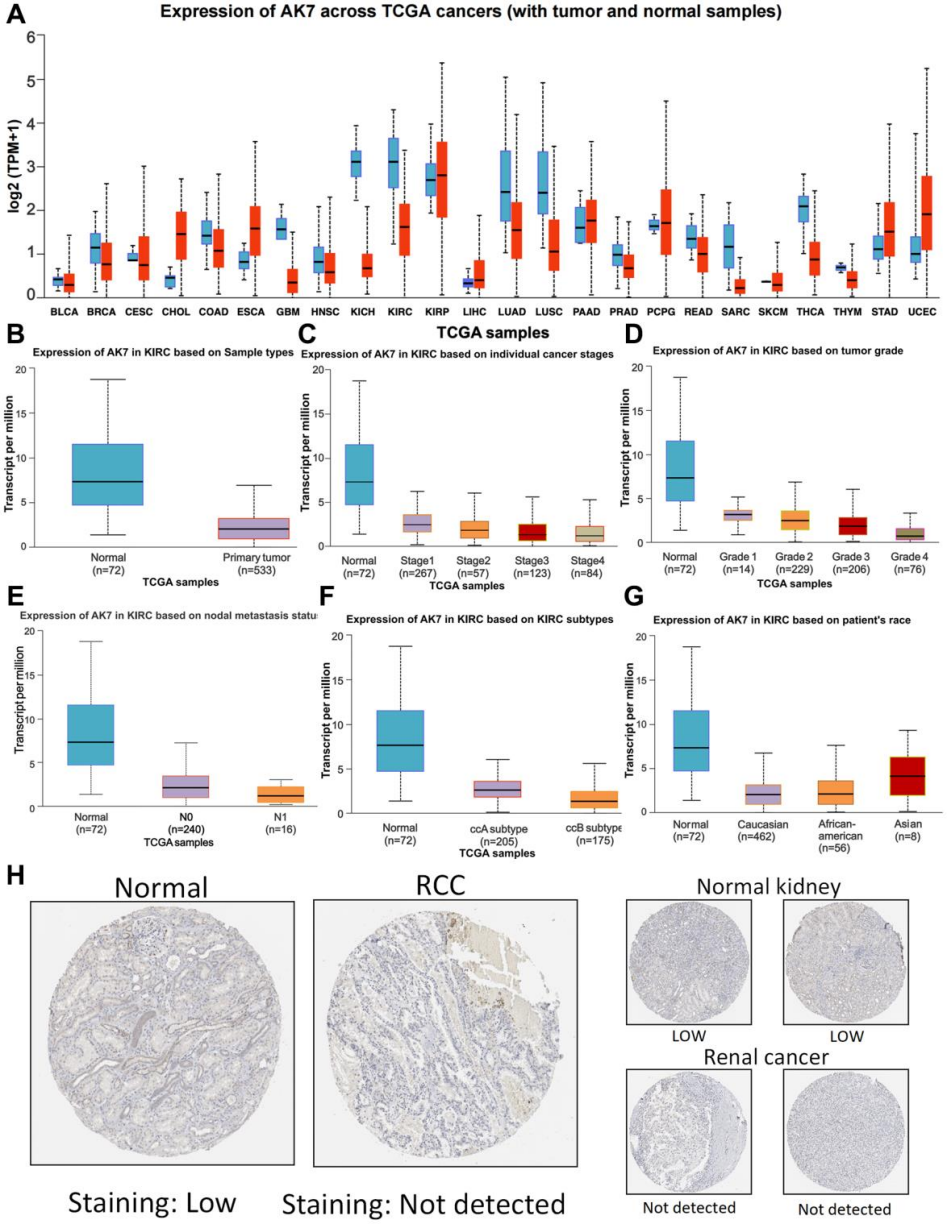
**Expression profile of AK7 in tumor.** (**A**) Expression of AK7 mRNA in different cancers and corresponding normal tissues. (**B**) The expression of AK7 mRNA in ccRCC was significantly higher than that in normal kidney tissue. (**C**–**G**) Differences in SPC25 mRNA expression depending on stage, grade, nodal metastasis status, subtype and race. (**H**) Expression of AK7 in normal renal tissues and ccRCC tissues. ^*^P < 0.05; ^**^P < 0.01; ^***^P < 0.001; ^****^P < 0.0001.

We initially utilized GTEx and UALCAN to explore the AK7 gene expression in both normal and tumor tissues. Our findings revealed great variations in the expression levels of the AK7 gene across different types of cancers. Specifically, we observed a substantial decrease in breast cancer, colon adenocarcinoma, glioblastoma multiforme, ccRCC and liver cancer compared to normal tissues. The levels of AK7 expression in bile duct carcinoma, esophageal carcinoma, gastric carcinoma, and endometrial carcinoma were found to be elevated compared to the levels in the respective ordinary tissues (Figure 1A). This observation indicates that AK7 expression exhibits strong tumor specificity. Consequently, our subsequent analysis primarily concentrated on examining AK7 expression in ccRCC. The findings indicated that the AK7 expression in cancer tissue was notably diminished (Figure 1B) and it seemed to decrease further with the advancement of ccRCC. Among them, AK7 expression was found to be the lowest in patients with stage 4 ccRCC (Figure 1C). A similar phenomenon was observed in those patients (Figure 1D). The expression of AK7 was reduced in individuals with lymph node metastasis compared to those without metastasis (Figure 1E). The aforementioned observations collectively indicated a correlation between the decreased expression of AK7 and the progression of the disease in individuals. Furthermore, the expression of AK7 was found to be lower in individuals with ccBRCC compared to ccARCC (Figure 1F). Additionally, a great disparity was noticed in AK7 expression between the Asian race and other races, with greater levels detected in the Asian population (Figure 1G). Further analysis of data from the Human Protein Atlas (HPA) confirmed the previous findings, demonstrating that AK7 expression in ccRCC was notably reduced (Figure 1H).

### AK7 knockdown promoted the proliferation, invasion, and migration ability of human ccRCC cell lines

**Figure 2:**
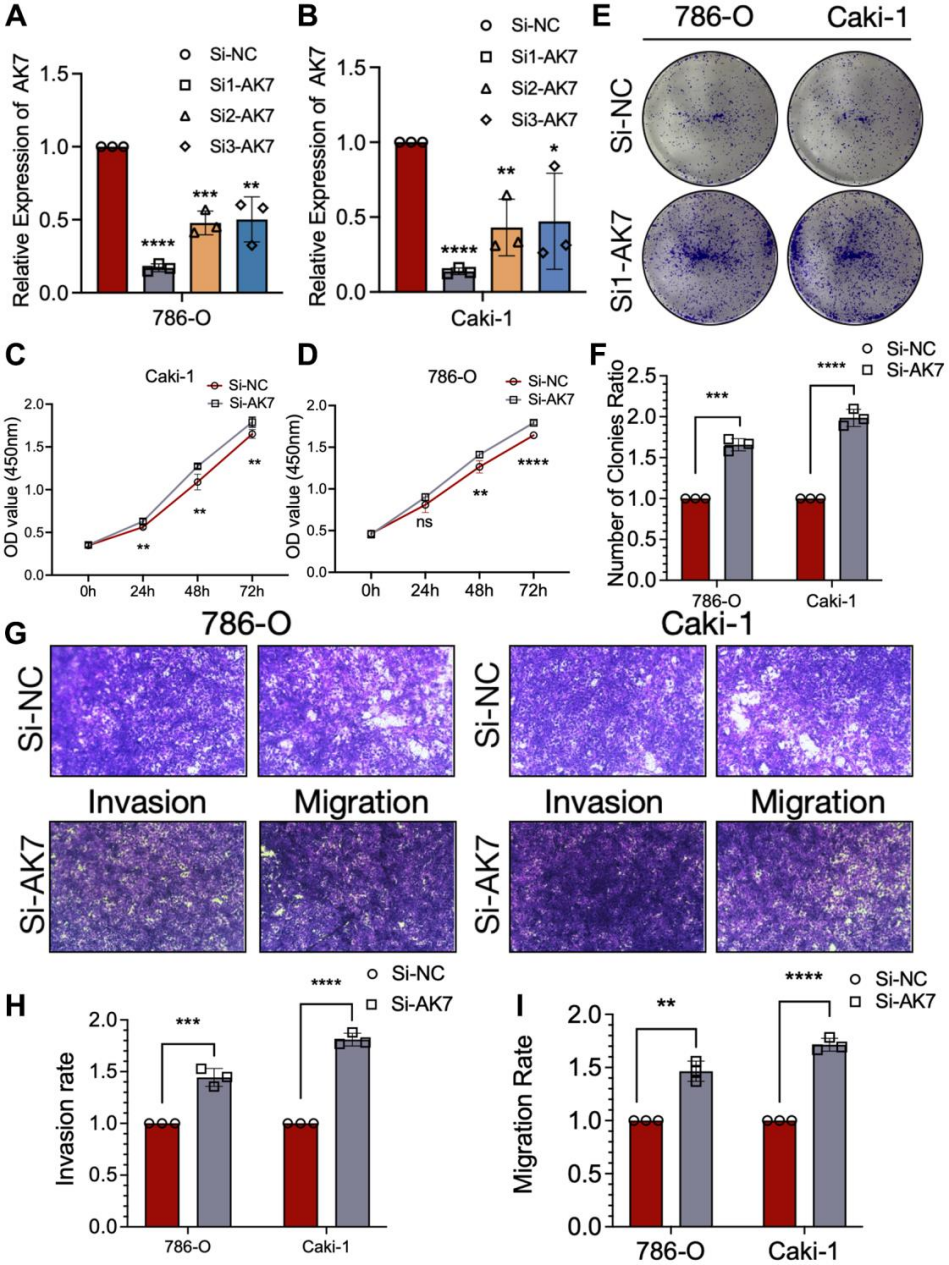
**AK7 knockdown promoted the proliferation, invasion and migration ability of human ccRCC cell lines.** (**A**, **B**) Three siRNAs (si1, si2, and si3) were designed to silence AK7 in ccRCC cells (786-O and Caki-1), and validated by qRT-PCR. (**C**, **D**) The growth curves of 786-O (**C**) and Caki-1 (**D**) cells were plotted after transfection with si1-AK7/si-NC based on CCK-8 assay. (**E**, **F**) Colony formation assays demonstrated that knockdown of AK7 promoted the proliferation of 786-O and Caki-1 cells. (**G**–**I**) Transwells experiment demonstrated that knockdown of AK7 expression could effectively promote the migration and invasion ability of ccRCC cells. ^*^P < 0.05; ^**^P < 0.01; ^***^P < 0.001; ^****^P < 0.0001.

To investigate the functional implications of AK7 in RCC, we developed three AK7-specific si-RNAs to knockdown AK7 expression in both cell lines. Following evaluation of knockdown efficiency using qRT-PCR, si1-AK7 exhibited the highest efficacy and was selected for subsequent experiments (Figure 2A, 2B). The findings from the CCK-8 assay demonstrated a significant enhancement in the growth ability of both cell lines following the knockdown of AK7 expression (Figure 2C, 2D). Comparable outcomes were noted in the colony formation experiments conducted on both cell lines (Figure 2E, 2F). We then evaluated migration and invasion capacity using the Transwell chamber. Following the knockdown of AK7, a marked increase in the infiltration and movement capabilities of the two cell lines was observed (Figure 2G–2I). These results suggested the potential antitumor activity of AK7 in ccRCC.

### Overexpression of AK7 suppressed the proliferation, invasion, and migration ability of human ccRCC cell lines

**Figure 3:**
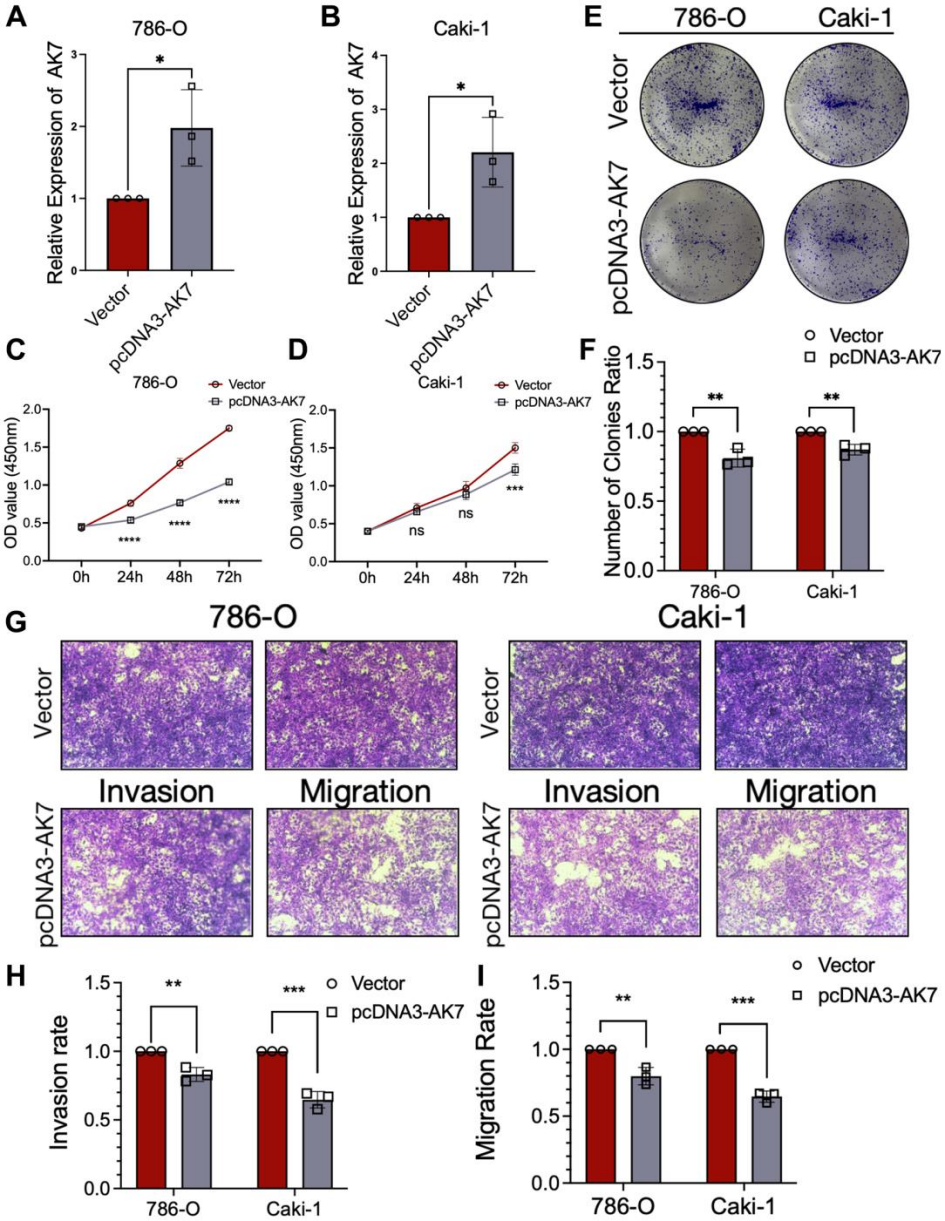
**Overexpression of AK7 inhibited proliferation, invasion and migration of human ccRCC cell lines.** (**A**, **B**) qRT-PCR verified the efficiency of overexpression of AK7 in 786-O and AKI-1 cell lines. (**C**, **D**) The growth curves of 786-O (**C**) and Caki-1 (**D**) cells were plotted after overexpression of AK7 based on CCK-8 assay. (**E**, **F**) Colony formation assays demonstrated that overexpression of AK7 inhibited the proliferation of 786-O and Caki-1 cells. (**G**–**I**) Transwells experiment demonstrated that overexpression of AK7 could effectively inhibit the migration and invasion ability of ccRCC cells. ^*^P < 0.05; ^**^P < 0.01; ^***^P < 0.001; ^****^P < 0.0001.

We constructed plasmids overexpressing AK7 to transfect 786-O cell lines and Caki-1 cell lines. The qRT-PCR findings demonstrated that transfection led to elevated AK7 expression in both cell lines (Figure 3A, 3B). The proliferation of CCK-8 cells revealed that the growth of ccRCC cell line declined significantly after AK7 overexpression (Figure 3C, 3D). Consistent findings were obtained from colony formation experiments conducted on the two cell lines, which revealed that AK7 overexpression facilitated cell proliferation (Figure 3E, 3F). Furthermore, Transwell assays revealed a great reduction in the infiltration and movement capabilities of both cell lines following AK7 overexpression (Figure 3G–3I). Altogether, these results provided further evidence of the tumor-inhibitory properties of AK7 expression.

### AK7 as a prognostic marker and predictive factor for immunotherapy efficacy in ccRCC individuals

**Figure 4:**
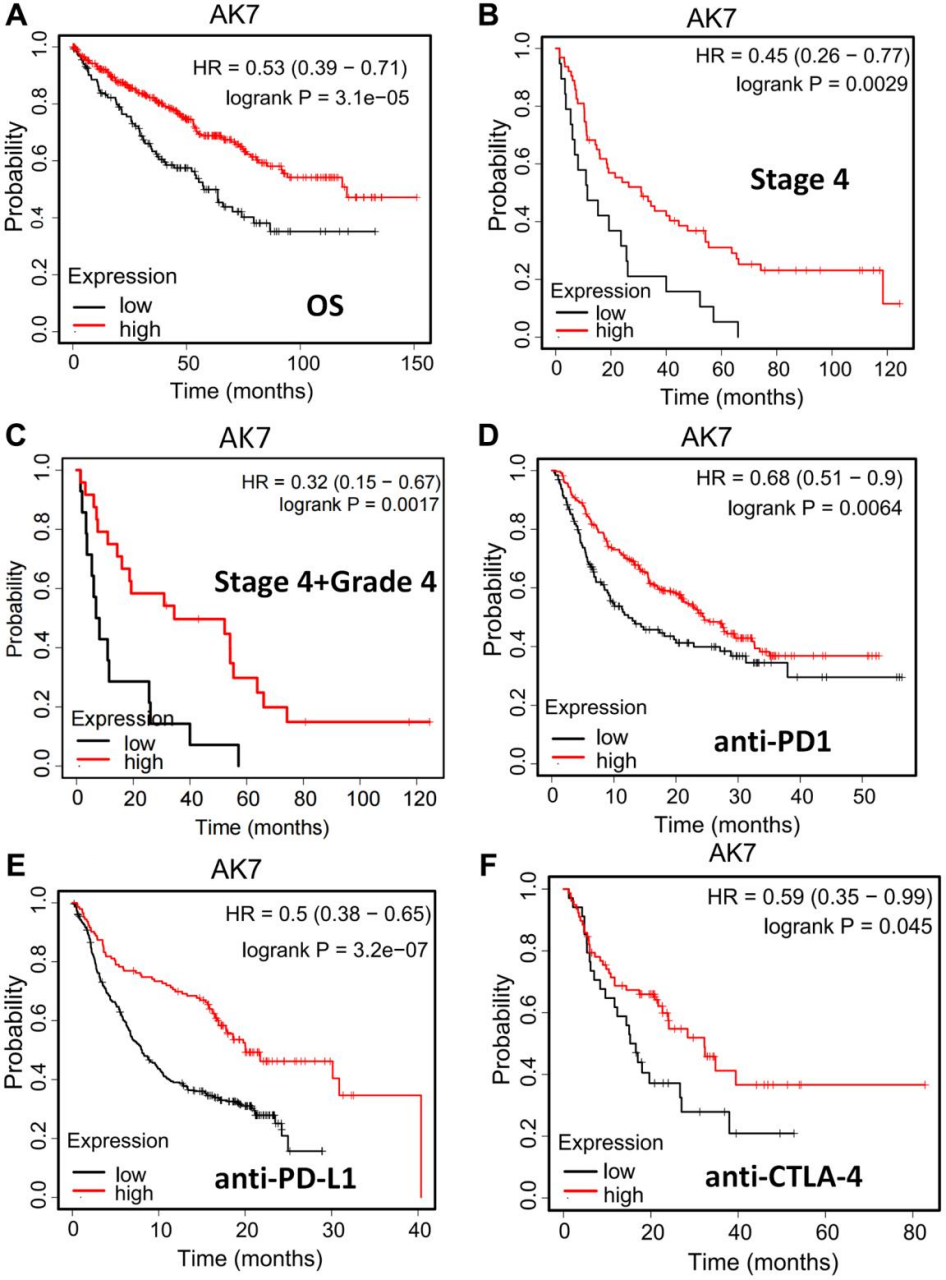
**AK7 can be used as a prognostic indicator and a predictor of immunotherapy effect in ccRCC patients.** (**A**) In pancarcinoma, patients with high expression of AK7 have a better prognosis than those with low expression. (**B**) In ccRCC, patients with high expression of AK7 had longer OS than those with low expression. (**C**) In ccRCC at stage 4, patients with high expression of AK7 had longer OS than those with low expression. (**D**–**F**) In patients treated with anti-PD1 (**D**), anti-PD-L1 (**E**), and anti-CTLA-4 (**F**), high expression of AK7 has a better prognosis. ^*^P < 0.05; ^**^P < 0.01; ^***^P < 0.001; ^****^P < 0.0001.

The connection between AK7 expression and individual prognosis was analyzed using the Kaplan-Meier plotter. The outcomes revealed that in pancarcinoma individuals, those with elevated AK7 expression had a more favorable prognosis than those with diminished expression (Supplementary Figure 1). A similar phenomenon was also observed in ccRCC patients (Figure 4A). Our analysis of TCGA data revealed a correlation between diminished AK7 expression and advanced tumor stages. Furthermore, our findings indicated that in ccRCC individuals at stage 4, those with diminished AK7 expression exhibited a greatly poorer prognosis compared to those with elevated expression (Figure 4B). Furthermore, this trend is even more pronounced in patients with grade 4, stage 4 (Figure 4C). These findings demonstrated that AK7 could hold promise as a possible indicator of future outcomes and may influence the effectiveness of immunotherapy. We are encouraged by the observation that patients with elevated AK7 expression display heightened responsiveness to immunotherapy. Individuals with elevated AK7 expression showed improved prognosis when treated with anti-PD1 (Figure 4D), anti-PD-L1 (Figure 4E), and anti-CTLA-4 (Figure 4F). The findings suggested that AK7 may serve as a possible indicator of future to enhance sensitivity to immunotherapy.

### The expression of AK7 was correlated with immunosuppressive factors

**Figure 5:**
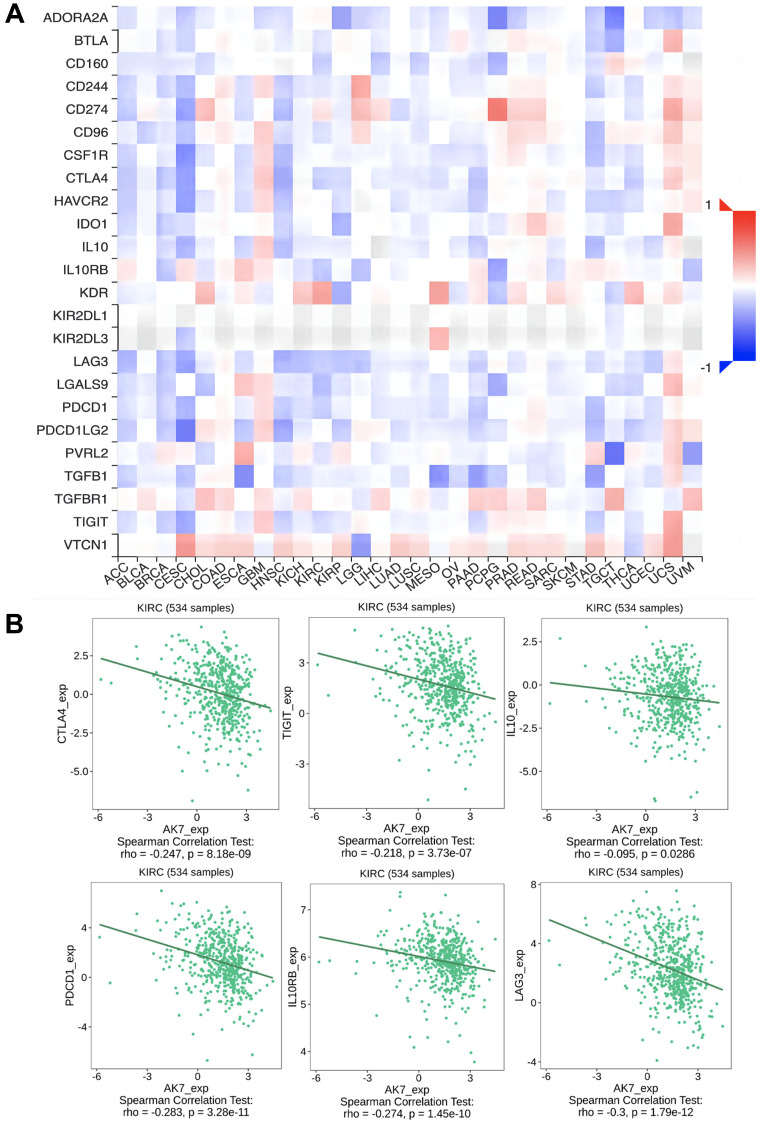
**The expression of AK7 was correlated with immunosuppressive factors.** (**A**) Correlation between AK7 and immunoinhibitors in different cancers. (**B**) The expression of AK7 was negatively correlated with the expression of CTLA4, TIGIT, IL10, PD1, IL0RB, LAG3 and other immunosuppressive factors. ^*^P < 0.05; ^**^P < 0.01; ^***^P < 0.001; ^****^P < 0.0001.

These results arouse our interest in exploring the role of AK7 in the immune response to cancer. We employed TISIDB, a comprehensive repository platform for collaborations between the immune system and tumors, to investigate the correlation between AK7 and diverse immune regulatory factors across different types of tumors (Figure 5A). Upon further examination, we were surprised to discover that AK7 expression indicated an inverse connection with the expression of several immunosuppressive factors. In ccRCC, CTLA4, TIGIT, IL10, PDCD1, LGA3 and other immunosuppressive factors were inversely connected with the expression of AK7 (Figure 5B). In addition, the TISIDB database showed that AK7 was correlated with the expression of multiple immune cell subtypes, immune stimulators, and MHC molecules (Supplementary Figures 2–4).

### Overexpression of AK7 inhibits RCC growth and enhances anti-PD1 efficacy

**Figure 6:**
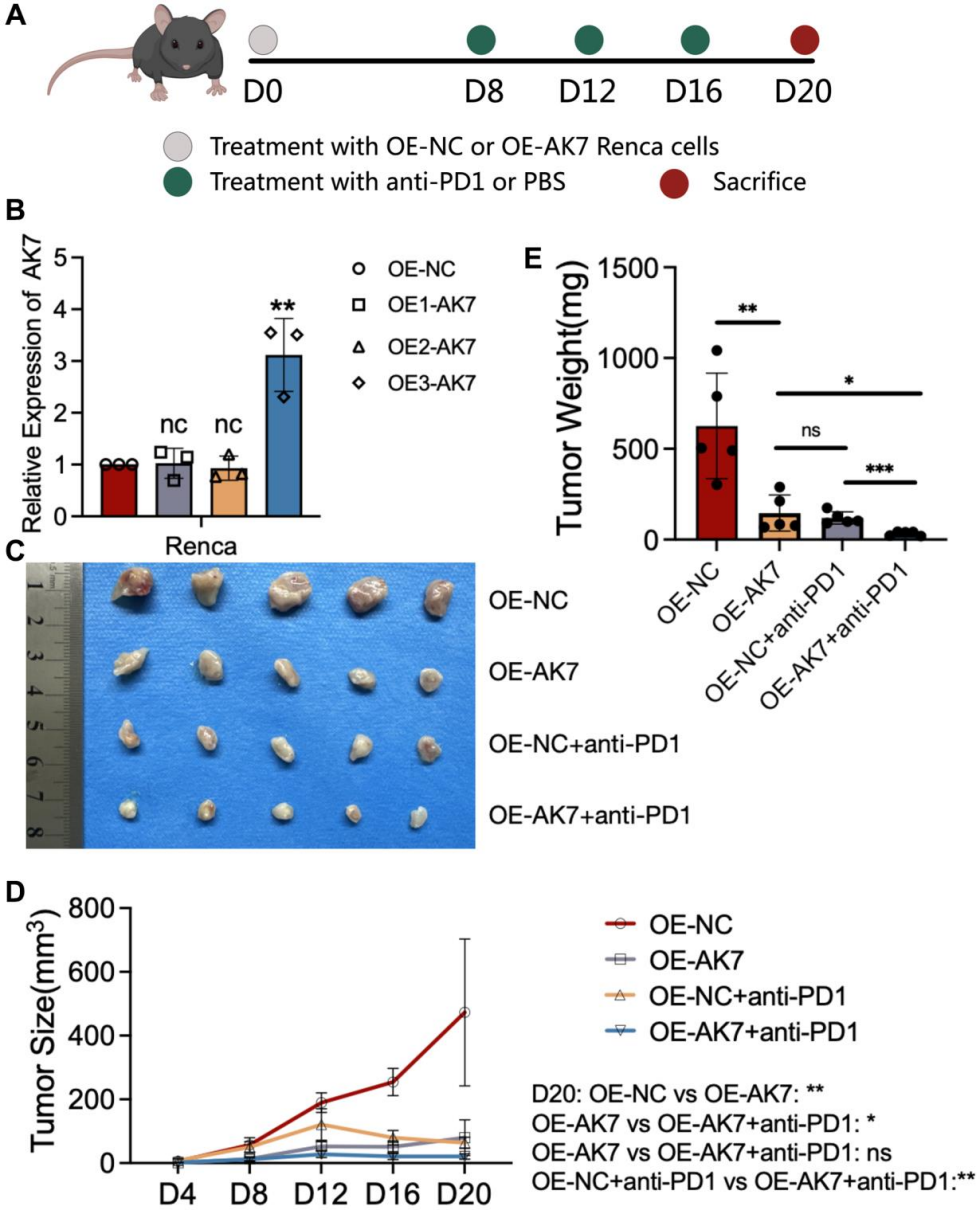
**Overexpression of AK7 inhibits RCC growth and enhances anti-PD1 efficacy.** (**A**) Schematic diagram of establishment of mouse subcutaneous tumor model in each group. (**B**) Three lentiviruses were designed to overexpress AK7 expression in RENCA cell lines, and their overexpression efficiency was verified by qRT-PCR. (**C**) Images of subcutaneous tumors in each group. (**D**, **E**) Analysis of subcutaneous tumors in the respective groups. ^*^P < 0.05; ^**^P < 0.01; ^***^P < 0.001; ^****^P < 0.0001.

To delve deeper into the impact of AK7 expression on immunotherapy, we constructed three AK7-targeting lentiviruses to upregulate AK7 expression in mouse RCC cell lines and conducted *in vivo* experiments in mice (Figure 6A). The qRT-PCR results indicated that OE3-AK7 had the highest overexpression efficiency (Figure 6B). Subsequently, we established a mouse subcutaneous tumor model using normal RENCA cells and AK7-overexpressing RENCA cells. The findings demonstrated a great deceleration in the tumor growth velocity in mice following AK7 overexpression. Furthermore, following AK7 overexpression, the tumor growth velocity of mice in the combined anti-PD1 set exhibited a great deceleration compared to the anti-PD1 set or the group with AK7 over-expression alone (Figure 6C–6E). These results once again demonstrated the potential anti-tumor and anti-PD1 sensitizing capabilities of AK7 targets.

## DISCUSSION

The majority of previous studies have primarily focused on exploring the role of AK7 in PCD and primary male infertility. Limited research has been conducted on elucidating the involvement of AK7 in tumor development. Consequently, only one study to date has examined the prognostic significance of AK7 in ovarian cancer. Our research indicated that AK7 is notably downregulated in various subtypes of ccRCC, and low AK7 expression is a predictor of unfavorable prognosis among individuals with this type of tumor. Therefore, we further explored the role of AK7 in ccRCC. Based on our cellular experiments, it was observed that downregulating AK7 expression in ccRCC cell line led to substantial enhancement in tumor growth, infiltration, and mobility. Increasing its expression could effectively inhibit the growth, infiltration, and mobility of ccRCC cell lines. To validate the role of AK7, we conducted additional experiments on animals. The outcomes confirmed that elevating the levels of AK7 in kidney cancer cell lines via lentivirus, led to a substantial reduction in the growth velocity of mouse tumors. These results unequivocally demonstrated the pivotal role of AK7 in the onset and progression of ccRCC. AK7 emerged as a promising target for therapy and a valuable marker for prognosis for ccRCC.

Cancer immunotherapy involves a variety of methods that aim to stimulate or enhance the body's immune system to specifically target cancer cells. Two of the earliest discovered checkpoints are known as CTLA4 and PD1. Immune checkpoints, such as those mentioned earlier, have the ability to diminish T cell activity and even suppress T cells through a series of mechanisms. ICIs currently in use, such as anti-PD1 and anti-PD-L1, work by blocking the activity of these checkpoints, reinvigorating the antitumor capabilities of T cells within tumors, and fundamentally reshaping immune function. Research findings indicated that about half of the cancer individuals experienced positive outcomes from ICIs like ipilimumab (anti-CTLA4) and nivolumab (anti-PD1) [37–39], which represents a promising advancement in treating cancer.

Numerous studies have documented the use of immunotherapy in treating RCC. This approach has greatly shifted the treatment paradigm for individuals with advanced RCC, changing the outcomes previously achieved with anti-angiogenic drugs that focus on the VEGF pathway. The U.S. Food and Drug Administration (FDA) gave its approval on November 23, 2015, for the use of nivolumab in treating aRCC individuals who have previously undergone drug treatment targeting vascular endothelial growth factor (VEGF) [40]. Moreover, the FDA cleared the use of a mix of immunotherapies of nivolumab and ipilimumab, in 2018 for treating aRCC individuals who are either at low-risk or easily treatable, and have not undergone any prior treatment. In 2019, the FDA authorized the use of pembrolizumab (anti-PD1) together with axitinib (VEGFR-TKI) and avelumab (anti-PD-L1) together with acitinib as primary treatment in aRCC individuals. Concurrently, ongoing research is focusing on further applications of ICIs in the treatment of RCC.

Despite its effectiveness, only a small portion of cancer patients experience a substantial and long-lasting response to PD1 blockade [41]. When compared to the conventional targeted therapy and cytotoxic chemotherapy, immunotherapy has a notably increased prevalence of drug resistance. Consequently, a large number of cancer individuals are unable to benefit from it, posing the most significant challenge for immunotherapy [42]. Resistance to PD1/PD-L1 antibody is linked to a range of cytokines produced by the tumor and metabolic pathways within the tumor [43]. In previous research, resilience has been divided to secondary resistance and primary resistance in accordance with the development of resistance. Secondary resistance happens when the initial use of PD1/PD-L1 mAb is effective, but resistance emerges as the treatment progresses. In contrast, primary resistance to drugs refers to the initial immunotherapy treatment's lack of effectiveness [44]. Previous studies have verified that medication resistance development is mainly associated with the expression of PD1/PD-L1 on the cell membrane. Moreover, additional crucial factors that contribute to drug resistance comprise impairment of primary and co-stimulatory indications, modifications in the TME and epigenetic alterations [45]. Here, we discovered a potential target, AK7, which could serve as an indicator for predicting the therapeutic reaction to anti-PD1, anti-PD-L1, and anti-CTLA-4 treatments. Individuals exhibiting high AK7 expression levels had a higher probability of respond positively to these immunotherapies and demonstrate improved prognosis. In addition, following the upregulation of AK7 expression in mouse renal cancer cell line RENCA using lentivirus, we established a subcutaneous tumor model and subsequently administered a combination treatment involving anti-PD1. The results suggested that increasing the enhancement of AK7 expression can substantially enhance the efficacy of PD1 blockade therapy. These findings suggested that AK7 served as not only a predictive target for immunotherapy-sensitive types but also a promising target for enhancing effectiveness of immunotherapy. This study explored AK7 as a possible predictor of prognosis and a target for therapy for ccRCC, as well as a predictive target for the therapeutic effect of anti-PD1 treatment. Enhancing the AK7 expression has the possibility to enhance the therapeutic effectiveness of anti-PD1. This study has certain limitations. We did not carry out a more in-depth verification of the role of AK7 in gene knockout mice or patient-derived xenograft (PDX) models, and we did not deeply explore the molecular mechanism associated with AK7 and PD1, which will be the focus of our future studies.

## Supplementary Materials

Supplementary Figures
