# Supplementary material for: AK7-deficiency reversal inhibits ccRCC progression and boosts anti-PD1 immunotherapy sensitivity
Source: Aging (Albany NY). 2024 Jul 5;16(13):11072–89. doi: 10.18632/aging.206006 (PMC11272107; doi:10.18632/aging.206006)
Supplement: Supplementary Figures [file aging-16-206006-s001.pdf]

## SUPPLEMENTARY FIGURES

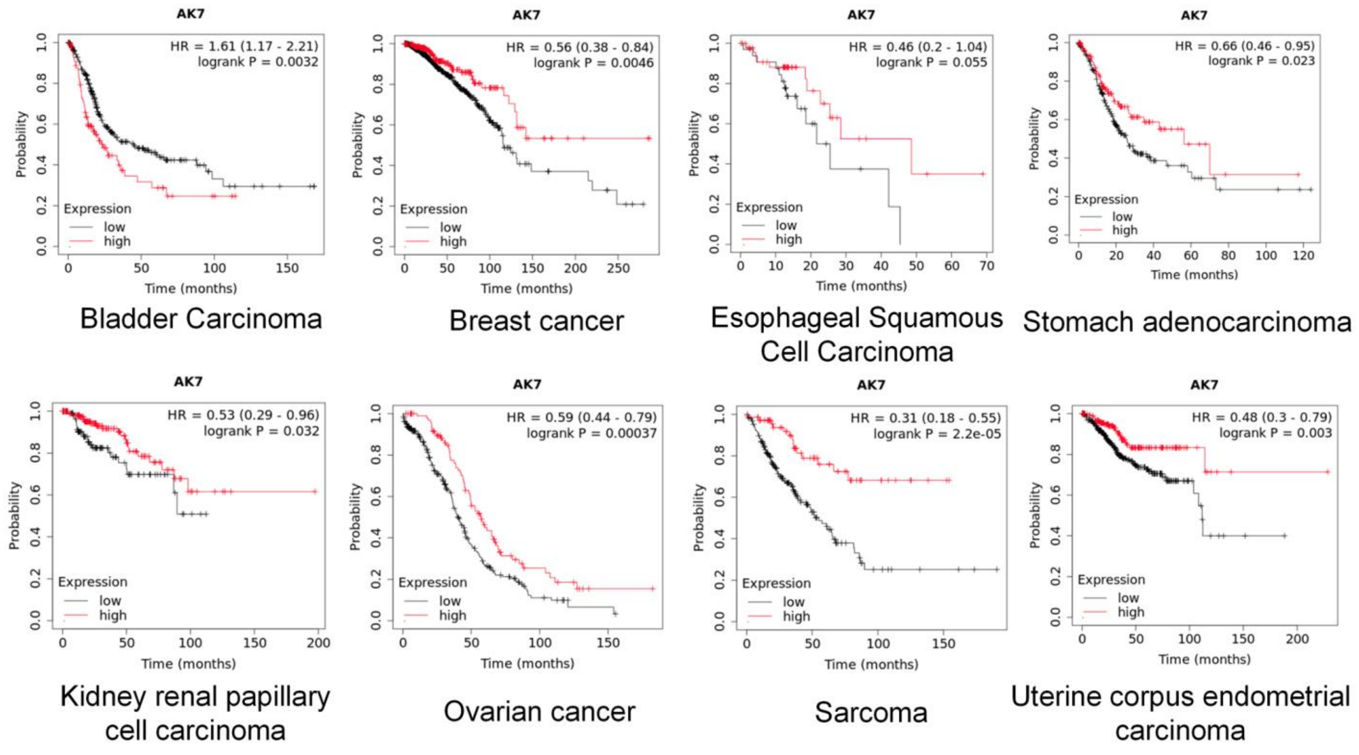


**Supplementary Figure 1. Relationship between AK and prognosis in different cancer species.**

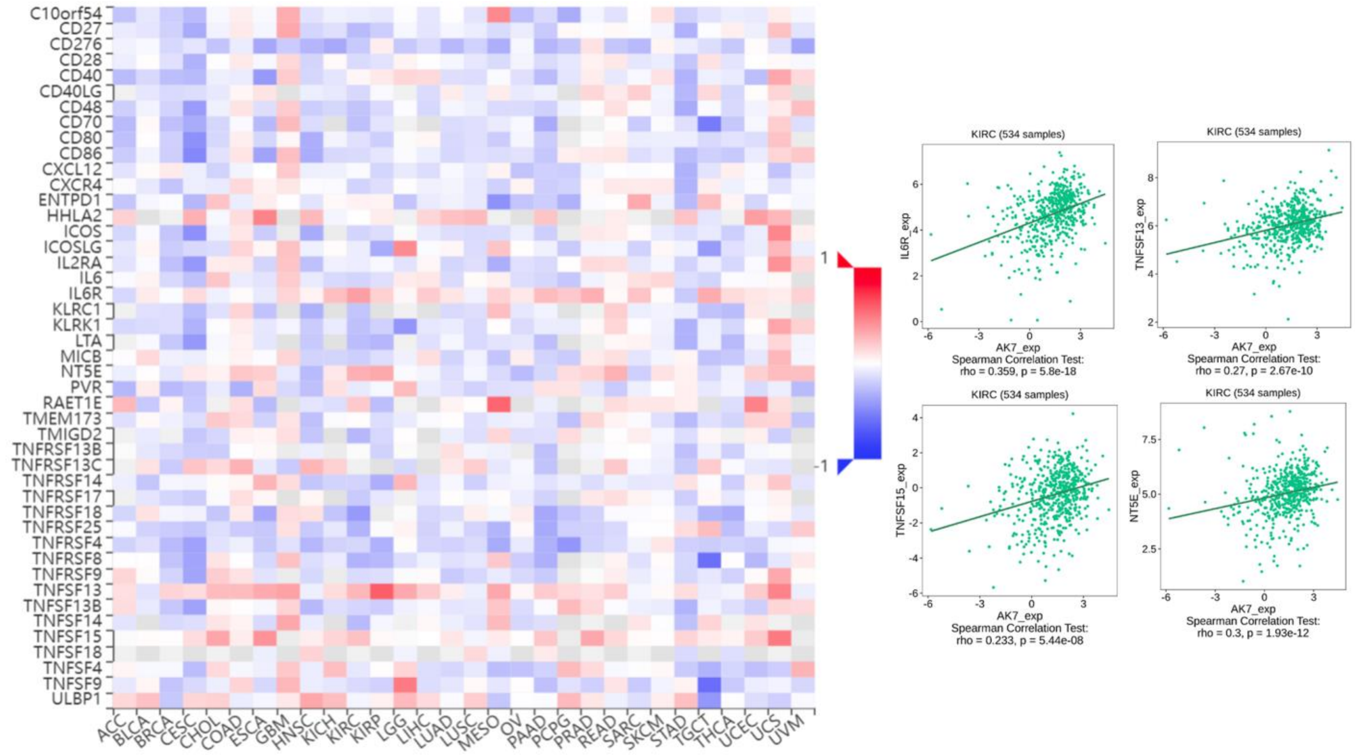


**Supplementary Figure 2. Spearman correlations between of AK7 and immunostimulators across human cancers.**

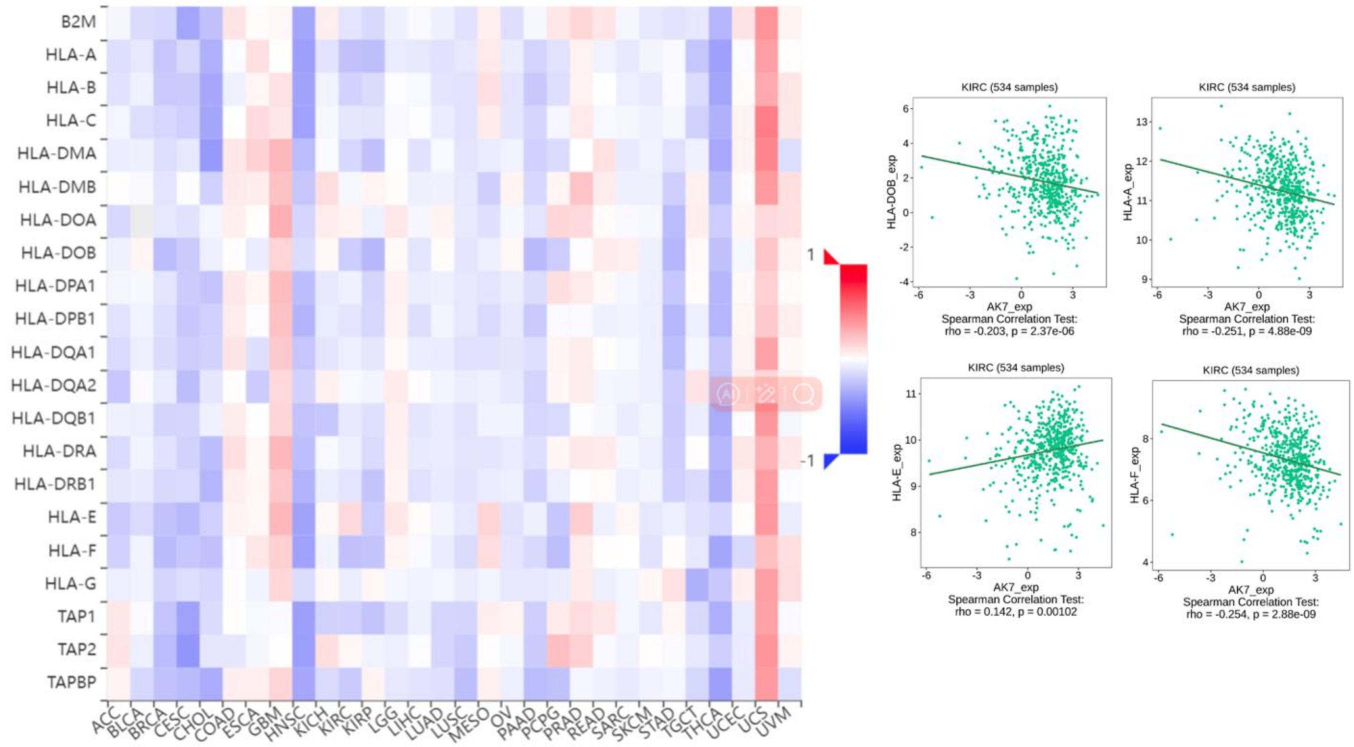


**Supplementary Figure 3. Spearman correlations between AK7 and MHCs across human cancers.**

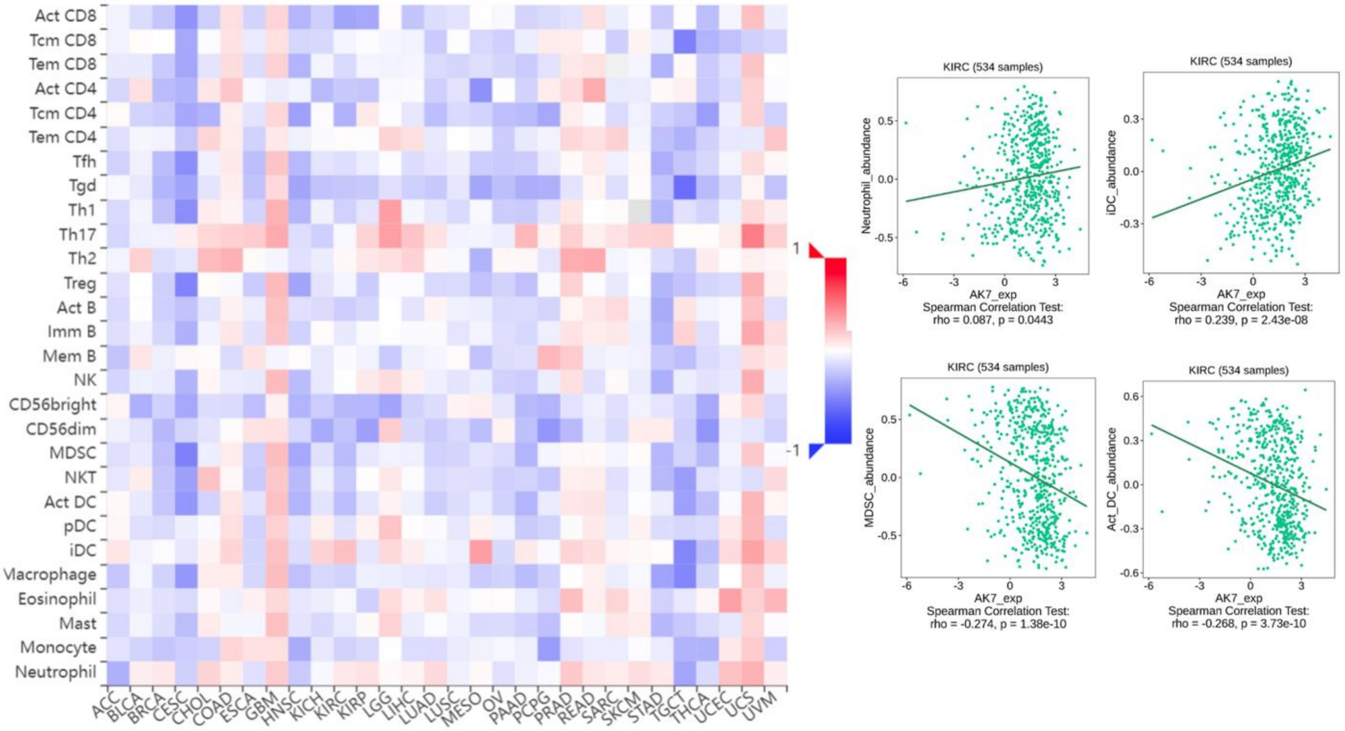


**Supplementary Figure 4. Spearman correlations between AK7 and TILs across human cancers.**
